# The Impact of Unplanned Excision on the Outcomes of Patients With Soft Tissue Sarcoma of the Trunk and Extremity: A Propensity Score Matching Analysis

**DOI:** 10.3389/fonc.2020.617590

**Published:** 2021-01-22

**Authors:** Yao Liang, Tian-Hui Guo, Bu-Shu Xu, Dong-Chun Hong, Hai-Bo Qiu, Zhi-Wei Zhou, Xing Zhang

**Affiliations:** ^1^ State Key Laboratory of Oncology in South China, Collaborative Innovation Center for Cancer Medicine, Sun Yat-sen University Cancer Center, Guangzhou, China; ^2^ Department of Gastric Surgery, Sun Yat-sen University Cancer Center, Guangzhou, China; ^3^ Melanoma and Sarcoma Medical Oncology Unit, Sun Yat-sen University Cancer Center, Guangzhou, China

**Keywords:** soft tissue sarcoma, trunk and extremity, oncologic outcomes, planned excision, unplanned excision

## Abstract

**Background:**

Unplanned excision (UPE) of soft tissue sarcoma (STS) is often chosen in the early phase by general physicians without any radiological evaluation.

**Purpose:**

The present study aimed to evaluate the impact of UPE on the clinical outcomes of patients with STS of the trunk and extremity.

**Materials and Methods:**

Patients with STS of the trunk and extremity who underwent R0 resection between 1998 and 2016 were included and divided into the UPE and planned excision (PE) groups. Propensity score matching (PSM) was used to control the selection bias. The endpoints were disease-specific survival (DSS), local recurrence-free survival (LRFS), and metastasis-free survival (MFS).

**Results:**

In total, 458 patients (277 males, 181 females; median age: 43 years) were included: 329 (71.8%) in the PE group and 129 (28.2%) in the UPE group. The follow-up time ranged from 7.1 to 313.78 months, with a median of 112.18 months. UPE patients were more likely to have a smaller or superficial lesion and were more frequently administered adjuvant therapy. After PSM, compared with the PE group, the UPE group had a longer LRFS (P=0.015), but there was no difference between the two groups regarding DSS and MFS. Residual disease was observed in 77.5% of the re-resected specimens in the UPE group and was a risk factor for DSS (P = 0.046) and MFS (P = 0.029) but was not associated with local recurrence (LR) (P=0.475) or LRFS (P=0.334). Moreover, we found no difference in DSS, LRFS or MFS according to the interval from UPE to definitive resection.

**Conclusion:**

STS treated with UPE had distinct characteristics. Patients who undergo UPE followed by an additional wide R0 resection have similar oncological survival compared to patients who undergo an initial PE, although the high incidence of residual tumor in the UPE group leads to an unfavorable clinical course.

## Introduction

Soft tissue sarcoma (STS) represents a rare and heterogeneous group of primary malignancies, accounting for approximately 1% of all adult malignancies (1). Since its incidence is low and it can occur in any part of the body, STS is apt to being ignored by general physicians and is inadvertently excised as a mass assumed to be benign or an inflammatory lesions without wide margins *(* 2). Approximately 19% to 53% of patients who undergo inappropriately unplanned excision (UPE) are referred to sarcoma centers (2–4 *). Due to the high risk of residual* tumor*s left in the tumor bed after UPE*, the current standard therapy suggests additional resection to achieve wide or at least negative margins and ensure local control (5, 6).

Several studies have demonstrated that patients who underwent UPE had worse oncologic outcomes (7, 8). whereas other authors showed similar or even better outcomes than patients who underwent planned excision (PE) initially (9–11). These differing results are probably because the majority of the literature did not consider similar clinicopathological variables. It is difficult to draw conclusions from two comparison groups with disparate tumor features even in multivariable models in which differences can partly be explained (12, 13). Thus, it is necessary to construct an algorithm to balance the differences in baseline characteristics between patients who undergo UPE and PE.

The present study is aimed to compare the oncologic outcomes of patients with STS of the trunk and extremity who underwent UPE with those of patients who underwent PE. We created a group from the study population with balanced baseline characteristics according to some primary characteristics through a propensity score matching (PSM) and then elucidate the potential influence of UPE. Additionally, we evaluated factors were associated with the prognosis and whether residual tumor or delayed re-excision would affect prognosis.

## Materials and Methods

### Study Population

We reviewed the STS database from the Sun Yat-sen University Cancer Center (SYSUCC, Guangzhou, China) to identify patients who underwent R0 resection as the final resection status for primary, non-metastatic STS of the trunk and extremity between January 1998 and January 2016. Patients with inadequate medical records (50, 7%) and those who were lost to follow-up (27 unreachable patients at the point of follow-up, 3%) were excluded. Patients with stage IV disease and those who underwent preoperative treatment were also excluded. Finally, 458 patients were included in this study (**Supplementary Figure 1**).

According to previous reports, UPE was defined as the non-oncologic excision of a suspected benign lesion without consideration the need to remove the normal tissue around the tumor with subsequently pathologically confirmed STS (14, 15). PE was defined as the planned oncologic excision for a preoperatively suspected STS. R0 was defined as the microscopic absence of malignant cells at the resection margin. All tumors were reviewed by experienced pathologists at our institution. Tumor size was determined as the largest diameter described in the pathology reports or measured on imaging, and sarcoma depth was characterized as superficial or deep according to the involvement of the investing muscle fascia. Tumors were staged and graded according to the American Joint Committee on Cancer (AJCC) 8th Edition (16) and the Fédération Nationale des Centres de Lutte Contre le Cancer (FNCLCC) grading system respectively (17).

After surgery, all patients were regularly followed-up every 3–6 months during the first 2–3 years and yearly thereafter. Recording of medical history, physical examination, computed tomography (CT) and/or magnetic resonance imaging (MRI) were performed during the follow-up. Additional studies, including positron emission tomography (PET) and biopsy were performed when necessary. The follow-up time was calculated from the date of diagnosis to the date of death or was censored at the end of follow-up (March 1st, 2020). The primary endpoints were disease-specific survival (DSS), local recurrence-free survival (LRFS), and metastasis-free survival (MFS). The time to the occurrence of the event was calculated from the date of R0 surgery to the date when the event was first recorded.

The authenticity of this article was validated by uploading the key raw data to the Research Data Deposit public platform (www.researchdata.org.cn) with the RDD approval number of RDDA2020001446. Our institutional review board (IRB) approved this study (B2020-068-01).

### PSM Analysis

The propensity score, defined as the conditional probability of undergoing a therapy given certain covariate factors of covariates, is generally calculated to adjust selection bias in observational studies (18, 19). In our study, one-to-one nearest-neighbour matching without replacement was adopted to control confounding factors in both groups using a 0.1 calliper. PSM was performed by using Empower Stats software (http://www.empowerstats.com/).

### Statistical Analysis

Chi-square tests (e.g., Fisher’s exact test and Pearson’s chi-square test) were used for comparisons of categorical data, where appropriate. Survival curves were generated by using the Kaplan-Meier method and compared by using the log-rank test. Prognostic variables associated with DSS, LRFS, and MFS that were significant in the univariate analyses were selected for multivariate Cox proportional hazard model analyses with the stepwise forward selection algorithm, and the results are presented as hazard ratios (HR) and 95% confidence intervals (CI). Two-sided *P* values < 0.05 were considered statistically significant. All data were analyzed using the IBM SPSS software, version 20.0 (SPSS, Inc., IBM Company, Armonk, New York).

## Results

### Baseline Clinicopathological Characteristics Prior to PSM

As shown in **Table 1**, there were 277 male patients and 181 female patients with a male: female ratio of 1.53:1. The mean age was 43 (25th–75th percentile: 31–55) years. Among the 458 patients, 129 patients (28.2%) underwent UPE while 329 patients (71.8%) underwent PE. The most common histological subtypes were fibrosarcoma (n=125, 27.3%) and undifferentiated pleomorphic sarcoma (n=107, 23.4%). A total of 45.6% of lesions were greater than or equal to 5 cm, and 57.0% were deep tumors. A total of 320 (69.9%) patients were histologically classified as G2/G3, 138 (30.1%) patients were classified as stage I, 194 (42.4%) as stage II, and 126 (27.5%) as stage III. Altogether, 27.3% of patients (n=125) underwent adjuvant treatment after surgery with chemotherapy (n=29, 6.3%), radiotherapy (n=77, 16.8%), or combined chemoradiotherapy (n=19, 4.1%).

**Table 1:** Baseline clinicopathological characteristics before and after propensity score matching.

Characteristics	Unmatched cases N = 458 (%)	Matched (1:1) cases N = 214 (%)
Sex		
Male	277 (60.5)	128 (59.8)
Female	181 (39.5)	86 (40.2)
Age at operation (years)		
<50	291 (63.5)	135 (63.1)
≥50	167 (36.5)	79 (36.9)
Body mass index (kg/m^2^)		
<18.5	48 (10.5)	26 (12.1)
≥18.5 to <25.0	289 (63.1)	131 (61.2)
≥25.0	121 (26.4)	57 (26.6)
Pathological types		
Fibrosarcoma	125 (27.3)	61 (28.5)
liposarcoma	66 (14.4)	26 (12.1)
Undifferentiated pleomorphic sarcoma/MFH	107 (23.4)	57 (26.6)
Leiomyosarcoma	13 (2.8)	5 (2.3)
Synovial sarcoma	62 (13.5)	21 (9.8)
Rhabdomyosarcoma	16 (3.5)	7 (3.3)
Alveolar soft part sarcoma	10 (2.2)	5 (2.3)
Angiosarcoma	4 (0.9)	3 (1.4)
Malignant peripheral nerve sheath tumor	28 (6.1)	14 (6.5)
Others	27 (6.9)	15 (7.0)
Tumor size (cm)		
<5	249 (54.4)	139 (65.0)
–10	175 (38.2)	62 (39.0)
>10	34 (7.4)	13 (6.0)
Tumor site		
Upper extremity	78 (17.0)	40 (18.7)
Lower extremity	202 (44.1)	88 (41.1)
Thoracic/trunk/ abdominal wall	178 (38.9)	86 (40.2)
Tumor depth		
Superficial	197 (43.0)	112 (52.3)
Deep	261 (57.0)	102 (47.7)
Tumor grade		
G1	138 (30.1)	53 (24.8)
G2	245 (53.5)	134 (62.6)
G3	75 (16.4)	27 (12.6)
Unplanned excision		
No	329 (71.8)	107(50.0)
Yes	129 (28.2)	107 (50.0)
Residual	100 (77.5)	83 (77.6)
No residual	29 (22.5)	24 (22.4)
AJCC stage		
IA	93 (20.3)	41 (19.2)
IB	45 (9.8)	12 (5.6)
II	194 (42.4)	104 (48.6)
IIIA	103 (22.5)	45 (21.0)
IIIB	23 (5)	12 (5.6)
Adjuvant therapy		
None	333 (72.7)	148 (69.2)
Chemotherapy	29 (6.3)	13 (6.1)
Radiotherapy	77 (16.8)	41 (19.2)
Combined chemoradiotherapy	19 (4.1)	12 (5.6)
Local Recurrence		
Yes	186 (40.6)	87 (40.7)
No	272 (59.4)	127 (59.3)
Metastasis		
Yes	90 (19.7)	42 (19.6)
No	368 (80.3)	172 (80.4)
Survival status		
Alive	351 (76.6)	162 (75.7)
Dead	107 (23.4)	52 (24.3)

AJCC, American Joint Committee on Cancer.

In addition, with a median follow-up of 112.18 months (range, 7.1–313.78 months), 107 patients (23.4%) died of STS, and the 5-year DSS rate was 86.9%. A total of 186 patients (40.6%) developed local recurrence (LR), of whom 115 were alive at the last follow-up. The median time to LR was 11 months. Distant metastasis (DM) occurred in 90 patients (19.7%), of whom 81 died during the follow-up. The median time to DM was 21 months.

### PE Compared With UPE

**Figure 1:**
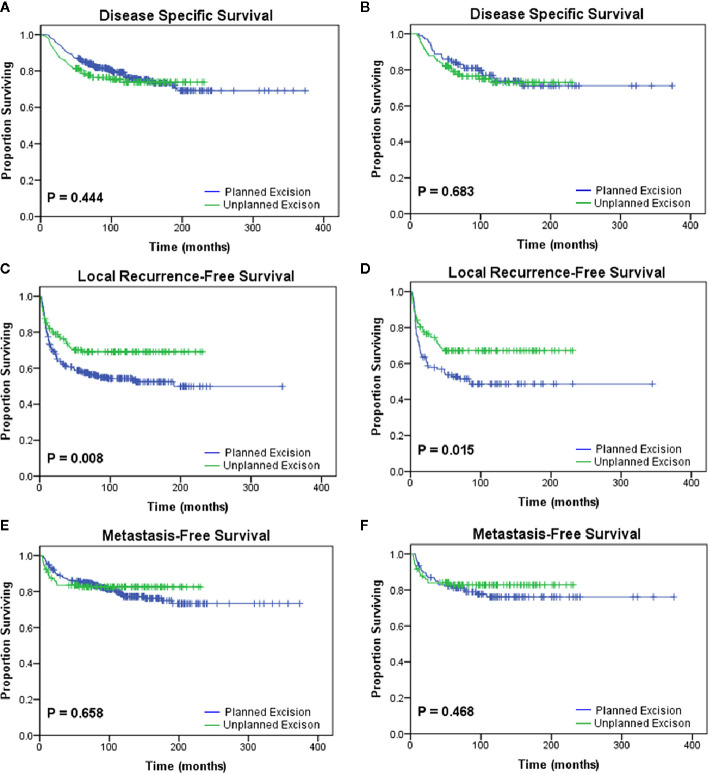
Kaplan-Meier analyses of the oncologic outcomes of the planned excision group and unplanned excision group in the unmatched study cohort **(A, C, E)** and the matched study cohort **(B, D, F)**. Patients who underwent unplanned excision followed by R0 resection had similar **(A, B)** disease-specific survival (p=0.444, p=0.683) and **(E, F)** metastasis-free survival (p=0.658, p=0.468) but improved **(C, D)** local recurrence-free survival (p=0.008, p=0.015) compared with those who underwent planned excision.

The PE and UPE groups did not differ in sex, age, body mass index (BMI), tumor location, DM, or death. However, compared with the PE group, the UPE group had a higher proportion of small-diameter lesions (<5 cm: 70.5% vs. 48.0%, P<0.001), lesions in superficial locations (55.8% vs. 38.0%, P=0.001), and adjuvant therapy administered (42.6% vs. 21.3%, P<0.001) and had different distributing trends for tumor grade (P=0.001) and AJCC stage (P=0.003). Based on Kaplan-Meier survival analysis, patients in the UPE group had better LRFS (P=0.008) but a lower 3-year LRFS rate (UPE 78% vs PE 89.1%, P=0.036) than those in the PE group. There was no difference in DSS (P=0.444) or MFS (P=0.658) between the two groups (**Figures 1A, C, E**; **Table 2**).

**Table 2:** Comparison of clinicopathological characteristics between planned excision (PE) group and unplanned excision group (UPE) before and after propensity score matching.

Characteristics	Unmatched (complete) dataset			Matched (1:1) dataset		
	PEN = 329	UPE N = 129	*P* Value	PE N = 107	UPE N = 107	*P* Value
Gender			0.674			0.577
male	197 (59.9)	80 (62.0)		62 (57.9)	66 (61.7)	
female	132 (40.1)	49 (38.0)		45 (42.1)	41 (38.3)	
Age (years)			0.523			0.321
<50	212 (64.4)	79 (61.2)		71 (66.4)	64 (59.8)	
≥50	117 (35.6)	50 (38.8)		36 (33.6)	43 (40.2)	
Body mass index (kg/m^2^)			0.962			0.778
<18.5	34 (10.3)	14 (10.9)		14 (13.1)	12 (11.2)	
≥18.5 to<25.0	207 (62.9)	82 (63.6)		63 (58.9)	68 (63.6)	
≥25.0	88 (26.7)	33 (25.6)		30 (28.0)	27 (25.2)	
Diameter (cm)			<0.001			0.667
<5cm	158 (48.0)	91 (70.5)		68 (63.6)	71 (66.4)	
≥5cm	171 (52.0)	38 (29.5)		39 (36.4)	36 (33.6)	
Tumor location			0.504			0.265
Trunk	131 (39.8)	47 (36.4)		47 (43.9)	39 (36.4)	
Extremity	198 (60.2)	82 (63.6)		60 (56.1)	68 (63.6)	
Tumor depth			0.001			0.784
Superficial	125 (38.0)	72 (55.8)		57 (53.3)	55 (51.4)	
Deep	204 (62.0)	57 (44.2)		50 (46.7)	52 (48.6)	
Tumor grade			0.001			0.545
G1	107 (32.5)	31 (24.0)		27 (25.2)	26 (24.3)	
G2	159 (48.3)	86 (66.7)		64 (59.8)	70 (65.4)	
G3	63 (19.1)	12 (9.3)		16 (15.0)	11 (10.3)	
AJCC stage			0.003			0.669
I	108 (32.8)	30 (23.3)		27 (25.2)	26 (24.3)	
II	123 (37.4)	71 (55.0)		49 (45.8)	55 (51.4)	
III	98 (29.8)	28 (21.7)		31 (29.0)	26 (24.3)	
Adjuvant treatment			<0.001			0.767
Yes	70 (21.3)	55 (42.6)		32 (29.9)	34 (31.8)	
No	259 (78.7)	74 (57.4)		75 (70.1)	73 (68.2)	
Recurrence			0.002			0.008
YES	148 (45.0)	38 (29.5)		53 (49.5)	34 (31.8)	
NO	181 (55.0)	91 (70.5)		54 (50.5)	73 (68.2)	
Metastasis			0.381			0.302
YES	68 (20.7)	22 (17.1)		24 (22.4)	18 (16.8)	
NO	261 (79.3)	107 (82.9)		83 (77.6)	89 (83.2)	
Status			0.832			1.000
Alive	253 (76.9)	98 (76.0)		81 (75.7)	81 (75.7)	
Dead	76 (23.1)	31 (24.0)		26 (24.3)	26 (24.3)	
5-year DSS	85.1%	76.6%	0.149	73.8%	75.0%	0.871
3-year LRFS	89.1%	78.0%	0.036	52.6%	73.4%	0.003
3-year MFS	90.8%	83.6%	0.134	78.9%	82.8%	0.354

PE, planned excision; UPE, unplanned excisions; AJCC, American Joint Committee on Cancer; DSS, disease specific survival; LRFS, local recurrence-free survival; MFS, metastases-free survival.

After PSM, two paired cohorts of 107 patients each were generated for both the UPE and PE groups where the baseline covariates (including size, depth, grade, AJCC stage, adjuvant therapy, etc.) were properly balanced. In the matched cohorts, the UPE group had a longer median LRFS (UPE 23.60 months vs. PE13.80 months, P=0.015) than the PE group, but no difference in DSS (P=0.683) or MFS (P=0.468) was found (**Figures 1B, D, F**). Additionally, UPE with subsequent R0 resection improved the 3-year local control rate of the tumor (3-year LRFS: UPE 73.4% vs. PE 52.6%, P = 0.003) compared with PE, which was exactly the opposite of the unmatched results. Oncological outcomes, including the 5-year DSS rate (PE 73.8% vs. UPE 75.0%, P = 0.871) and 3-year MFS rate (PE 78.9% vs. UPE 82.8%, P = 0.354), were not significantly different between the two groups (**Table 2**).

### Predictive Factors for Oncologic Outcomes

In the univariate analysis, tumor size, tumor depth, tumor grade, and AJCC stage were prognostic factors for DSS, LR, and DM (**Table 3**). Multivariate analysis demonstrated that tumor size and tumor grade remained independent predictors for both DSS (P=0.007, P<0.001) and DM (P=0.031, P< 0.001), but the resection status (UPE or PE) was not related to the DSS or DM. It is also worth noting that receiving adjuvant treatment was independently associated with worse DSS (with vs. without: HR 0.64, 95% CI: 0.43–0.94, P = 0.022) and an increased risk of DM (with vs. without: HR 0.54, 95% CI: 0.36–0.82, P = 0.004). Subsequent analysis revealed that location in the trunk (P = 0.020), lower tumor grade (P = 0.001), and receiving UPE (P = 0.027) were independent protective factors for LR (**Table 4**).

**Table 3:** Univariate analyses of variables for disease specific survival, local recurrence and distant metastases (unmatched complete datasets).

Variables	Disease specific survival		Local recurrence		Distant metastases	
	HR (95% CI)	*P* Value	HR (95% CI)	*P* Value	HR (95% CI)	*P* Value
Sex		0.786		0.535		0.197
Male	1 (referent)		1 (referent)		1 (referent)	
Female	0.95 (0.64–1.40)		0.68 (0.64–1.23)		0.75 (0.48–1.16)	
Age (years)		0.971		0.042		0.062
<50	1 (referent)		1 (referent)		1 (referent)	
≥50	0.99 (0.69–1.48)		1.35 (1.01–1.81)		0.64 (0.40–1.02)	
Tumor size (cm)		<0.001		0.047		0.003
<5	1 (referent)		1 (referent)		1 (referent)	
≥5	2.06 (1.40–3.04)		1.34 (1.00–1.78)		1.91 (1.25–2.91)	
Tumor location		0.091		0.001		0.221
Trunk	1 (referent)		1 (referent)		1 (referent)	
Extremity	1.42 (0.95–2.14)		1.70 (1.24–2.32)		1.32 (0.85–2.04)	
Tumor depth		<0.001		<0.001		<0.001
Superficial	1 (referent)		1 (referent)		1 (referent)	
Deep	2.42 (1.57–3.72)		1.77 (1.31–2.41)		2.37 (1.48–3.78)	
Tumor Grade		<0.001		<0.001		<0.001
G1	1 (referent)		1 (referent)		1 (referent)	
G2	4.12 (2.05–8.31)		1.71 (1.18–2.47)		11.98 (3.75–38.25)	
G3	11.22 (5.42–23.25)		2.92 (1.90–4.49)		26.11 (7.96–85.72)	
AJCC stage		<0.001		<0.001		<0.001
IA + IB	1 (referent)		1 (referent)		1 (referent)	
II	4.11 (2.01–8.41)		1.58 (1.08–2.31)		11.72 (3.63–37.78)	
IIIA + IIIB	7.72 (3.80–15.66)		2.44 (1.65–3.62)		19.67 (6.11–63.37)	
Unplanned excision		0.444		0.009		0.658
Yes	1 (referent)		1 (referent)		1 (referent)	
No	0.85 (0.56–1.29)		1.61 (1.13–2.30)		1.12 (0.69–1.80)	
Adjuvant therapy		<0.001		0.726		<0.001
Yes	1 (referent)		1 (referent)		1 (referent)	
No	0.48 (0.33–0.70)		0.94 (0.68-1.30)		0.39 (0.26-0.59)	

HR, hazard ratio; CI, confidence interval; AJCC, American Joint Committee on Cancer.

**Table 4:** Multivariate analyses of variables for disease specific survival, local recurrence and distant metastases (unmatched complete datasets).

Variables	Disease specific survival		Local recurrence		Distant metastases	
	HR (95% CI)	*P* value	HR (95% CI)	*P* value	HR (95% CI)	*P* value
Tumor size (cm)		0.007				0.031
<5	1 (referent)				1 (referent)	
≥5	1.72 (1.16–2.56)				1.60 (1.05–2.46)	
Tumor location				0.020		
Trunk			1 (referent)			
Extremity			1.46 (1.06–2.01)			
Tumor depth				0.050		
Superficial			1 (referent)			
Deep			1.37 (1.00–1.90)			
Tumor Grade		<0.001		0.001		<0.001
G1	1 (referent)		1 (referent)		1 (referent)	
G2	3.64 (1.80–7.39)		1.61 (1.10–2.36)		10.19 (3.17–32.73)	
G3	8.88 (4.23–18.60)		2.32 (1.48–3.62)		20.10 (6.08–66.67)	
Unplanned excision				0.027		
Yes			1 (referent)			
No			1.52 (1.05–2.20)			
Adjuvant therapy		0.022				0.004
Yes	1 (referent)				1 (referent)	
No	0.64 (0.43–0.94)				0.54 (0.36–0.82)	

HR, hazard ratio; CI, confidence interval.

### Subgroup Analysis of the UPE Group

**Figure 2:**
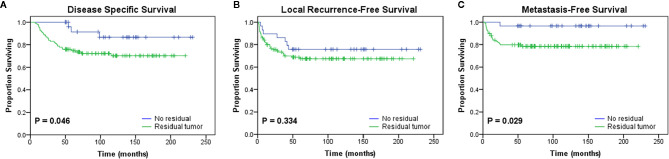
Kaplan-Meier curves showing the **(A)** disease-specific survival (p = 0.046), **(B)** local recurrence-free survival (p = 0.334), and **(C)** metastasis-free survival (p = 0.029) of patients after initial unplanned excision based on the presence or absence of residual tumor in the re-excision specimen.

Patients who underwent UPE were divided into the residual group (RG, n=100, 77.5%) and the no residual group (NRG, n=29, 22.5%) according to the presence or absence of macroscopic or microscopic tumors in the re-excised specimens. The RG showed significantly worse outcomes than the NRG in terms of DSS (P=0.046) and MFS (P=0.029) (**Figure 2**). Moreover, the residual tumor was associated with increased rates of DM (NRG 3.4% vs. RG 21%, P=0.026), but the trend towards higher mortality and LR rate were not statistically significant (**Table 5**).

**Table 5:** Oncologic outcomes for the unplanned excision subgroups.

	Time to definitive surgery			Residual		
	Short (1–30days) N = 56	Long (≥30days)N = 73	*P* Value	No N = 29	Yes N = 100	*P* Value
Survival status			0.822			0.053^a^
Alive	42 (75.0)	56 (76.7)		26 (89.7)	72 (72.0)	
Dead	14 (25.0)	17 (23.3)		3 (10.3)	28 (28.0)	
Local recurrence			0.329			0.475
No	37 (66.1)	54 (74.0)		22 (75.9)	69 (69.0)	
Yes	19 (33.9)	19 (26.0)		7 (24.1)	31 (31.0)	
Distant metastases			0.795			0.026^a^
No	47 (82.1)	60 (83.9)		28 (96.6)	79 (79.0)	
Yes	9 (17.9)	13 (16.1)		1 (3.4)	21 (21.0)	

^a^Fisher’s exact tests.

**Figure 3:**
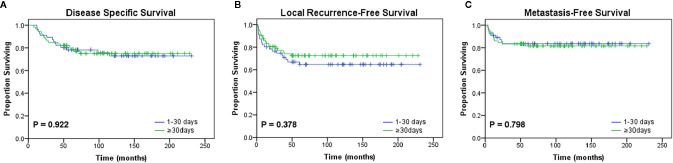
Kaplan-Meier curves for oncologic outcomes according to the interval from unplanned excision to definitive surgery. No differences in **(A)** disease-specific survival (p = 0.922), **(B)** local recurrence-free survival (p = 0.378), or **(C)** metastasis-free survival (p = 0.798) were observed between the two groups divided by the median interval value (30 days).

Additionally, the median time interval between UPE and re-excision was 30 days (range, 4 to 136 days; interquartile range, 22–43 days). To investigate the effect of a delayed re-resection on the study end points, the patients were divided into two cohorts: the short-interval group (<30 days) and the long-interval group (≥30 days) according to the median value. We observed no significant difference in prognosis between the two groups (**Figure 3**; **Table 5**).

## Discussion

This study was designed to investigate the impact of unplanned, non-oncologic excision on the outcomes of patients with STS of the trunk and extremity, with particular attention to similar baseline characteristics. To date, there is no consensus with regard to the potential survival effects of UPE. A recent retrospective study by Munoz et al. (20) reported that the risk of LR and DM was higher in patients who underwent re-resection than in those who underwent planned primary surgery. Worse oncologic outcomes were also reported by Qureshi et al. (7) and Saeed et al. (8) where the UPE group had worse LRFS and progression-free survival. These results might ascribe the poor prognosis in the UPE group to the residual tumor cells contained in muscular or fascial boundaries or fragmented excision (21). However, the findings of subsequent studies were inconsistent with the above conclusions and demonstrated similar or even better LRFS, MFS, and DSS in patients who underwent re-excision than in those with a planned definitive cancer resection as the primary surgical procedure (9, 11, 13, 22). This contradiction in results can be caused by the difficulty in comparing patients who undergo UPE with patients who undergo PE due to the more favorable tumor features and better biological characteristics (such as smaller size, more superficial location, and more benign) of patients with UPE. Additionally, some studies included the patients with various treatments after UPE, including re-excision, observation, and radiation, which affects the conclusions that can be reached (7). We believe that the conclusions are more convincing if the outcomes are compared between the two groups based on similar baseline characteristics.

Therefore, in our study, we used the PSM approach for possible confounding factors, which makes the analyses more precise. Compared to other relevant studies (9, 20, 21, 23), the patients included in our study were more likely to have smaller tumor size, and fibrosarcoma and undifferentiated sarcoma were the most common types of histology in our study. Based on our analysis, we found that UPE followed by R0 resection was associated with a better LRFS and 3-year LRFS rates than PE. According to our univariable and multivariable analyses, UPE decreased the risk of local recurrence and was confirmed as an independent predictive factor of LR. The risk of LR for PE patients was 1.52 times higher than that for UPE patients. Moreover, the UPE was not associated with worse DSS or a high risk of DM. More optimistic than our findings, Bianchi et al. (24) reported that UPE had a better sarcoma-specific survival and higher LR- and DM-free rates, which was likely driven by the complete re-excision after UPE. The above findings imply that for patients who undergo UPE, subsequent definitive oncologic re-excision is able to result in an acceptable outcome. This is of great value in areas with inadequate medical knowledge and poor technology. Unexpectedly, we also showed that adjuvant therapy was considered a significant contributing factor for death and metastasis. One reason may be that only patients with more aggressive tumors would choose postoperative treatment, and these patients had an inherently poor prognosis and were prone to metastasis. The highly malignant and invasive biology may significantly dilute the effectiveness of adjuvant therapy.

The present study revealed that 77.5% of patients had residual tumors in re-excised specimens (RTRS), which is within the range of 43% to 83% found in the literature (4, 24–26), supporting the significance of additional wide resection for patients undergoing UPE. Our results also showed that the RG presented worse oncological outcomes, including DSS and MFS, and the presence of RTRS was a risk factor for DM. However, we did not confirm a connection between the RTRS and LR, which was not in line with conventional understanding and several previous studies demonstrating that patients with RTRS were inclined to have shorter LRFS (14, 27–29). This might be attributable to the small sample size, since there were only 29 patients in the NRG in the present study. Our cohort was too small to reach statistical significance and further studies are needed to confirm this trend. Moreover, Han et al. (30) found that there were no difference in the prognosis and oncologic outcomes according to the time until definitive resection, which was in accordance with our results. Based on these findings, we support the view that any effect of delayed definitive surgery is likely to be of minor clinical significance (31).

Our study also shows that patients who underwent UPE were more likely to have a smaller or superficial lesion and were more often administered adjuvant therapy than those who underwent PE. These results showed that clinicians often adopted UPE strategies for lesions with good biological features. And in the traditional concept, the patients with UPE may have a worse prognosis than the patients underwent PE. Considering that these sarcoma patients underwent UPE, postoperative treatments were more inclined to be performed to reduce the potential “adverse” effects of UPE, even though the conditions were not that serious. In addition, over the past several decades, the postoperative adjuvant therapy of PE patients in China was non-standard, and the doctors did not determine adjuvant treatments recommendations based on standard pathologic factors of patients. Thus, the proportion of the UPE group receiving postoperative treatments is relatively higher than the PE group in our study. Therefore, since completely avoiding the occurrence of UPE in patients with STS is impractical, we recommend three principles of diagnosis and treatment to prevent deteriorated conditions from occurring: (a) identify and diagnose tumors based on the clinical history and imaging manifestations carefully before the initial surgery, having more awareness of the possibility of malignancy in lesions with a small size and superficial location; (b) emphasize the importance of eliminating the whole lesion completely and achieving negative margins initially; and (c) perform reoperation with a multidisciplinary approach as a salvage measure after UPE has occurred, regardless of the interval between UPE and re-excision. Certainly, considering the remaining high incidence of UPE, more widespread recognition of such an inadequate procedure is needed.

There are some limitations to our study. First, in this research, a relatively high LR rate was found (n=186, 40.6%), which was slightly higher than that in other relevant studies (32). Possible explanations are as follows: we included a cohort of patients with a long follow-up time (some for over 18 years), based on which the risk for LR was observed to increase accordingly. Nearly 70% of the enrolled patients in this study had an advanced tumor stage (II-III) or high grade (G2-G3) at the time of initial diagnosis. In fact, over a decade ago, many patients did not seek treatment until their limbs were heavily swollen, and doctors were often their last choice. Furthermore, due to a lack of awareness of the guidelines by general surgeons and the lack of standardized management, the resection margin rarely meets the requirements of the extended resection, and the proportion of patients receiving adjuvant treatment remained relatively low (27.3%), especially chemoradiotherapy (4.1%). Second, as a retrospective study, there was intrinsic selection bias (i.e., loss to follow-up and clinical decisions made based on the economic condition of the patients). However, for natural reasons, UPE cannot be studied prospectively. Third, this was a single-center study; therefore, the characteristics of the enrolled patients and the results of this study may not be generalizable to other populations, but our results still offer a good description of patients referred after UPE of sarcoma excision and serve as valuable references. Furthermore, we used PSM analysis to reduce bias. However, the small numbers in the matched dataset might impact the statistical probabilities of our results. Therefore, our conclusions should be verified in a larger population of STS patients from multiple centers.

In conclusion, STS treated with UPE had distinct characteristics, including smaller lesion sizes, superficial location, more benign features, and high risk of residual tumor. Our propensity score data provided evidence that, compared with PE, UPE followed by R0 resection has a major impact on local control and could result in comparable long-term oncologic outcomes in patients with STS of the trunk and extremity. Considering the large number of cases with residual disease found in re-excised specimens with residual disease, which is an unfavorable factor associated with the prognosis, a definitive salvage reoperation with multidisciplinary treatments should be performed for STS patients with UPE, regardless of the time interval.

## Data Availability Statement

The data that supports the findings of this study are available from the corresponding author upon reasonable request.

## Ethics Statement

The studies involving human participants were reviewed and approved by Institutional Review Board of Sun Yat-sen University Cancer Center. Written informed consent for participation was not required for this study in accordance with the national legislation and the institutional requirements.

## Author Contributions

YL, Z-WZ, and XZ conceived and designed the study. YL, T-HG, and B-SX collected the data. YL, T-HG, B-SX, D-CH, and H-BQ performed data analysis and interpretation. YL, T-HG, and B-SX contributed to writing and review and editing. All authors contributed to the article and approved the submitted version.

## Funding

This study was supported by a grant from the National Natural Science Foundation of China (no. 81902736).

## Conflict of Interest

The authors declare that the research was conducted in the absence of any commercial or financial relationships that could be construed as a potential conflict of interest.

## Supplementary Material

Supplementary Figure 1 Flow chart of the exclusion and inclusion criteria.
