# Supplementary figures and images for: The Impact of Unplanned Excision on the Outcomes of Patients With Soft Tissue Sarcoma of the Trunk and Extremity: A Propensity Score Matching Analysis
Source: Front Oncol. 2021 Jan 22;10:617590. doi: 10.3389/fonc.2020.617590 (PMC7862753; doi:10.3389/fonc.2020.617590)

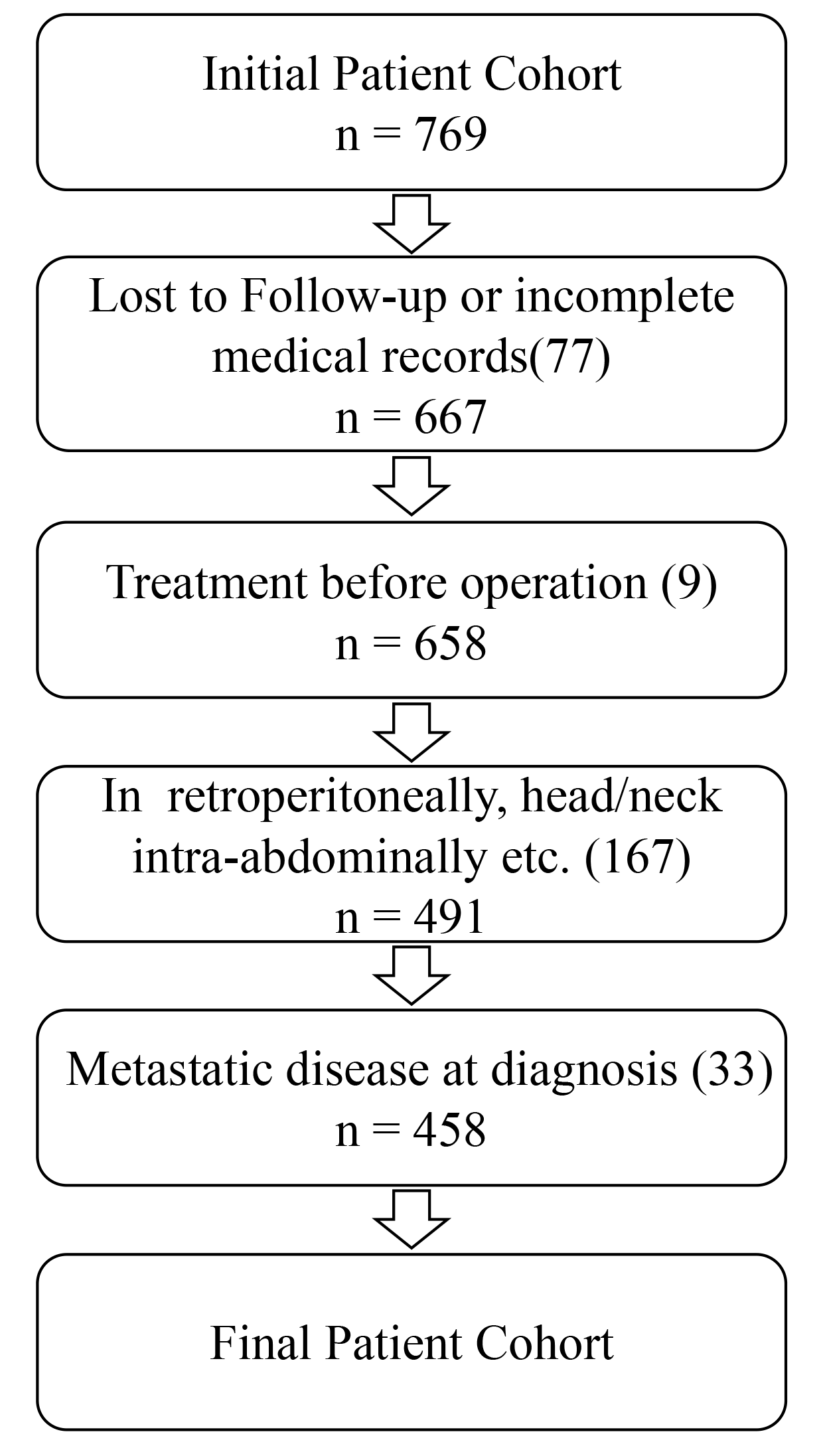

Supplement: Supplementary Figure 1 — Flow chart of the exclusion and inclusion criteria. [file Image_1.tif]
